# Estimating the rates of crossover and gene conversion from individual genomes

**DOI:** 10.1093/genetics/iyac100

**Published:** 2022-06-30

**Authors:** Derek Setter, Sam Ebdon, Ben Jackson, Konrad Lohse

**Affiliations:** Institute of Evolutionary Biology, University of Edinburgh, Edinburgh EH9 3FL, UK; Institute of Evolutionary Biology, University of Edinburgh, Edinburgh EH9 3FL, UK; Institute of Evolutionary Biology, University of Edinburgh, Edinburgh EH9 3FL, UK; Institute of Evolutionary Biology, University of Edinburgh, Edinburgh EH9 3FL, UK

**Keywords:** gene conversion, crossover, recombination

## Abstract

Recombination can occur either as a result of crossover or gene conversion events. Population genetic methods for inferring the rate of recombination from patterns of linkage disequilibrium generally assume a simple model of recombination that only involves crossover events and ignore gene conversion. However, distinguishing the 2 processes is not only necessary for a complete description of recombination, but also essential for understanding the evolutionary consequences of inversions and other genomic partitions in which crossover (but not gene conversion) is reduced. We present heRho, a simple composite likelihood scheme for coestimating the rate of crossover and gene conversion from individual diploid genomes. The method is based on analytic results for the distance-dependent probability of heterozygous and homozygous states at 2 loci. We apply heRho to simulations and data from the house mouse *Mus musculus castaneus*, a well-studied model. Our analyses show (1) that the rates of crossover and gene conversion can be accurately coestimated at the level of individual chromosomes and (2) that previous estimates of the population scaled rate of recombination $\rho =4N_{e}r$ under a pure crossover model are likely biased.

## Introduction

Genetic recombination, the exchange of genetic material between homologous chromosomes during meiosis, is one of the fundamental evolutionary processes. By creating novel combinations of alleles, recombination increases the efficacy of positive selection (Hill and Robertson 1966) and reduces the fitness burden of deleterious variants (Charlesworth and Charlesworth 1997). Recombination breaks down linkage disequilibrium (LD) in the genome and so determines the physical scale over which selective events interfere which each other and affect linked neutral sites (Charlesworth *et al.* 1993; Simonsen and Churchill 1997). Since recombination modulates virtually all evolutionary processes, understanding how and why it varies between organisms and between different regions of the genome remains a topic of intense research (see Stapley *et al.* 2017; Peñalba and Wolf 2020, for recent reviews). Beyond interest in recombination rate variation per se, estimates of recombination are also relevant for other inferences from genomic data. In particular, the power of quantitative or population genetic analyses depends crucially on recombination. Thus, while association studies or inference about past selection (e.g. DeGiorgio *et al.* 2016, 2014; Campos and Charlesworth 2019; Setter *et al.* 2020) and demography (Gutenkunst *et al.* 2009) often treat single nucleotide polymorphisms (SNPs) as independent for the purpose of obtaining point estimates, they rely on parametric bootstrapping or resampling procedures that are conditioned on a model of recombination to quantify uncertainty.

Recombination occurs via double-strand breaks which are either Holliday-junction mediated, resulting in crossovers (CO) and CO gene conversion (GC) events, or synthesis-dependent strand-annealing mediated, resulting in non-CO GC events (Resnick 1976; Szostak *et al.* 1983; Nassif *et al.* 1994). In a CO event, 2 nonsister chromatids break during pairing and reciprocally exchange sequence regions on either side of the break point (Griffiths *et al.* 2002). In contrast, GC, which typically occurs due to mismatch errors during replication (Carpenter 1982), involves the nonreciprocal copying of a short stretch of sequence, the GC tract (typically tens to hundreds of bases), from one nonsister chromatid to the other (Szostak *et al.* 1983; McMahill *et al.* 2007). The ratio of GC to CO rates varies widely across the tree of life: estimates range from 4 to 15× in humans (Jeffreys and May 2004) and mice (Li *et al.* 2019) to 1/2–1/10× in yeast, algae, and plants (Liu *et al.* 2018). Similarly, estimates of GC tract lengths range from 10 to several thousand base pairs between taxa (Casola *et al.* 2010; Mansai *et al.* 2011). Furthermore, the ratio of CO and GC may also vary drastically along the genome. In particular, CO may be severely reduced in centromeric and telomeric regions and within chromosomal inversions, while rates of GC may be unchanged (Talbert and Henikoff 2010; Korunes and Noor 2017) or even increased (Crown *et al.* 2018). Not only have early investigations into patterns of LD in humans highlighted that models of recombination without GC are insufficient at explaining fine-scale patterns of genetic variation (Przeworski and Wall 2001; Ptak *et al.* 2004), and there is reason to expect that GC may be the dominant force in deconstructing LD at an intragenic level (Andolfatto and Nordborg 1998). However, given that joint estimates for the rates of GC and CO within genomes and across taxa are sparse, the evolutionary causes and consequences of variation in these 2 components of recombination remain poorly understood.

Much effort has been devoted to estimating CO and GC directly from lab crosses (Hilliker *et al.* 1994), pedigrees (Kong *et al.* 2002; Johnston *et al.* 2016), or sperm-typing (Jeffreys and May 2004) data. However, such direct estimates are time consuming and expensive given that data from many meiotic events are required. While some pedigree-based (Smeds *et al.* 2016) and sperm-typing methods distinguish CO and GC events, most direct estimates of recombination are necessarily limited to CO events (Kong *et al.* 2002, 2014; Ma *et al.* 2015; Johnston *et al.* 2016). Since individual GC tracts are undetectable unless they span variants, the resolution to detect GC events is inherently limited by the scale of SNP variation.

Given the limitations of direct approaches for estimating recombination, methods that infer recombination indirectly from patterns of LD in whole-genome resequence data from natural populations are attractive. LD-based estimators of recombination implemented in popular tools such as *LDhat* (McVean *et al.* 2002; Auton and McVean 2007) and *LDhelmet* (Chan *et al.* 2012) are based on analytic expectations for pairs of loci which, given a large number of pairwise observations, can be used to compute the composite likelihood (CL) of the population-scaled rate of recombination $\rho =4N_{e}c$. However, current LD-based approaches for inferring recombination are limited in at least 2 ways.

First, both *LDhat* (McVean *et al.* 2002; Auton and McVean 2007) and *LDhelmet* (Chan *et al.* 2012) assume a simple model of recombination that only considers CO and ignores GC. Notable exceptions include the work of Gay *et al.* (2007) who extend the copying model of Li and Stephens (2003) to coestimate CO and GC rates, and Yin *et al.* (2009) who coestimate GC rates and tract lengths using hidden Markov models on SNP data. Second, since 2-locus approaches are generally conditioned on variant sites, they require phased data from many samples. Such data are still only available for a small minority of taxa. A recent exception *pyrho* is a phase-independent 2-locus method, however, to date this approach does not model GC (Spence and Song 2019). Additionally, there are several phase-independent methods alternative to the 2-locus approach for estimating recombination rates, such as using deep learning (Adrion *et al.* 2020) or the sequentially Markovian coalescent model to infer recombination maps (Barroso *et al.* 2019).

Here, we address both these limitations by developing a simple CL method that allows coestimation of CO and GC rates from individual diploid genomes. The calculation is based on analytic expectations for observing heterozygosity at 2 loci under the simplest model of recombination (CO only) and genetic drift (Strobeck and Morgan 1978; Lohse *et al.* 2011) and has previously been implemented by Haubold *et al.* (2010). We first use coalescent simulations to demonstrate that GC biases estimates of the CO rate and show that this bias depends on the physical distance between loci. By exploiting this nonlinear dependence of ρ on distance, we incorporate GC into the 2-locus expectations and build a framework for coestimating the rates of CO and GC. We apply our method to genome-wide data from wild-caught individuals of the house mouse *Mus musculus castanaeus*, compare our estimates to previous estimates of recombination based on a CO only model (Booker *et al.* 2017), and investigate the extent to which the rates of CO and GC are correlated with each other and with chromosome length.

We quantify the precision and accuracy of our estimates for *M.m. castaneus* using 100 parametric bootstrap simulations in msprime 1.0 (Baumdicker *et al.* 2022), and we use further simulations to test for robustness to violations of the underlying model. First, we investigate the ability of heRho to obtain an *average* estimate when there is underlying fine-scale recombination rate variation, as complex recombination landscapes have been demonstrated to reduce the reliability of LD-based inference methods (Raynaud *et al.* 2022). Second, we test for robustness to nonequilibrium population dynamics which are known to be problematic for methods that estimate recombination rates (Johnston and Cutler 2012; Kamm *et al.* 2016; Dapper and Payseur 2018). We consider 4 demographic models: (1) population size bottlenecks, (2) exponential growth, (3) historical admixture events, and (4) population substructure.

## Materials and methods

### Analytic expectation of 2-locus heterozygosity

We extended the models of Strobeck and Morgan (1978) and Haubold *et al.* (2010) to account for GC and use a CL approach to coestimate the rates of CO and GC and the mean GC tract length from individual genomes. We consider a neutral Wright–Fisher model for the evolution of 2 linked loci separated by *d* nucleotides in a population of *N* diploid individuals. Mutations occur at per-base rate *μ*. CO occurs at per-base rate *c* and results in an exchange of genetic material between sister chromatids. GC initiates at per-base rate *g* and GC tracts are replaced by the sequence from the sister chromatid. For the analysis, we rescale time by 1/2N generations and use the population-scaled parameters θ=4Nμ, κ=4Nc, and γ=4Ng for mutation, CO, and GC rates, respectively. We follow Wiuf (2000) in assuming that the GC tract length is an exponentially distributed random variable with mean *L* base pairs (Hilliker *et al.* 1994; Wiuf and Hein 2000).

The heterozygosity at a single site *H* with $E[H]=\frac{\theta }{1+\theta }\approx \theta$ is informative only about the depth of a local genealogy: a site is more likely to be heterozygous when the time to the most recent common ancestor, *T*_mrca_, is large and homozygous (i.e. identical in state) when *T*_mrca_ is small. Consider a second site at a fixed distance *d* and define *H*_0_, *H*_1_, and *H*_2_ as the proportion of all such pairs where neither site, one site, or both sites are heterozygous (respectively). These 2-locus measures of heterozygosity are informative about the joint distribution of the 2 underlying genealogies and allow estimation of the rate of recombination (Haubold *et al.* 2010; Lohse *et al.* 2011).

Using Eq. 4 of Strobeck and Morgan (1978), Haubold *et al.* (2010) derive analytic expressions for the expected frequency of *H*_0_, *H*_1_, and *H*_2_ as a function of ρ, the total rate of events which lead to recombination between 2 sites separated by *d* base pairs, agnostic to the underlying contributions of CO and GC:

(1) $$E_{\theta ,\rho }[H_{0}]=\frac{1}{{\left(1+\theta \right)}^{2}}+\Delta \frac{\theta }{{\left(1+\theta \right)}^{2}},$$

(2) $$E_{\theta ,\rho }[H_{2}]=\frac{\theta^{2}}{{\left(1+\theta \right)}^{2}}+\Delta \frac{\theta }{{\left(1+\theta \right)}^{2}}$$

(3) $$E_{\theta ,\rho }[H_{1}]=1-E_{\theta ,\rho }[H_{0}]-E_{\theta ,\rho }[H_{2}],$$

where

$$\Delta =\frac{\theta (18+\rho +18\theta +\rho \theta +4\theta^{2})}{18+13\rho +\rho^{2}+54\theta +40\theta^{2}+8\theta^{3}+\rho (\rho \theta +19\theta +6\theta^{2})}$$

represents the *zygosity* correlation (Lynch 2008) or the deviation from independence due to linkage. For large *d*, the genealogies at the 2 sites become independent, so $E_{\theta ,\rho }[H_{0}]=E{\left[H\right]}^{2}$ and $E_{\theta ,\rho }[H_{2}]={\left(1-E[H]\right)}^{2}$, the first term in Equations (1) and (2). In contrast, if the second site is tightly linked, the 2 sites likely share the same genealogy and we expect to see an excess in *H*_0_ and *H*_2_. As *d* increases, so too does the probability that recombination occurs between the sites, resulting in differing genealogies and an increase in *H*_1_.

### Coestimating crossover and gene conversion rates

The above expectations for *H*_0_, *H*_1_, and *H*_2_ make no assumption about the nature of recombination between pairs of sites, and ρ represents the rate at which the alleles transfers to different genetic backgrounds. For sites separated by a given distance *d*, we can obtain a maximum likelihood estimate for the total rate of recombination observed over these distances. Let $n_{d,0},\;n_{d,1}$, and $n_{d,2}$ be the counts of pairs in the genome corresponding to *H*_0_, *H*_1_, and *H*_2_ for a given distance *d*. The log-likelihood is then

(4) $$\text{ln}\;L_{d}(\rho ,d)=n_{d,0}\text{ln}(E_{d}[H_{0}])+n_{d,1}\text{ln}(E_{d}[H_{1}])+n_{d,2}\text{ln}(E_{d}[H_{2}]).$$

If we assume that recombination between the 2 loci occurs only through CO, recombination always transfers alleles onto different genetic backgrounds, and the per-base recombination rate ρ/bp is constant. This is not true for GC, because the 2 sites will still share a genealogy if the GC tract both initiates and terminates between them. In other words, recombination through GC occurs only if the GC tract spans only one of the 2 focal sites, in which case GC has the same effect as a CO event. Accounting for the probability of recombination during GC (Wiuf 2000; Wiuf and Hein 2000), we can rewrite the total rate of recombination ρ as a function of distinct rates of CO (κ) and GC (γ) and the expected GC tract length *L*(Langley *et al.* 2000; Frisse *et al.* 2001).

(5) $$\rho =\kappa d+2\gamma L\left(1-e^{-\frac{d}{L}}\right).$$

Given the dependence on distance *d*, observations $n_{d,0},\;n_{d,1}$, and $n_{d,2}$ for a single *d* are insufficient to estimate a 3-parameter model of recombination. However, by compositing the likelihood over many distances and substituting Equation (5) into Equations (1)–(3), we can coestimate the rate of CO κ, the rate of GC *γ*, and the mean tract length *L*.

The CL is thus given by

(6) $$\text{ln}\;CL(\kappa ,\gamma ,L)=\sum_{d_{\text{min}}}^{d_{\text{max}}}lnCL_{d}(\kappa ,\gamma ,L)$$

We have implemented the CL estimation described above in python as a simple open source tool, heRho which is available at https://github.com/samebdon/heRho. It is possible to estimate κ, γ, and *L* for 1 chromosome in approximately 10 s (with the estimation itself taking ≈0.2 s). The time to run the estimation step increases rapidly with the number of chromosomes up to approximately 10 min for 5 chromosomes or 1 day for the full analysis described below.

### Estimating recombination rates in the eastern house mouse

As a proof of principle, we tested our CL estimation of recombination on whole-genome data from a well-studied model species, the eastern house mouse *M. m. castaneus*. Both direct and indirect estimates for the total rate of recombination exist for this species (Booker *et al.* 2017) and several studies provide estimates for GC tract lengths (Paigen *et al.* 2008; Mansai *et al.* 2011; Cole *et al.* 2014; Li *et al.* 2019).

The data—originally described in Halligan *et al.* (2010) (ENA accession number PRJEB2176)—consists of Illumina (PE) resequence data for 10 individuals sampled from a wild *M. m. castaneus* population in India. Variant calling is described in Booker *et al.* (2021).

To minimize potential biases arising from background selection and the effect of selection on nearby linked sites, all analyses were restricted to intronic regions, which are putatively neutral. Specifically, we considered all introns >1 kb.

The final dataset included a total of 123,488 introns on autosomes 1–19, spanning a total of $9\times 10^{8}$ bases. For each intron, the positions of heterozygous sites in each individual were converted into 2-locus counts $n_{d,0},\;n_{d,1}$, and $n_{d,2}$ for each distance *d* included as part of heRho (https://github.com/samebdon/heRho). heRho obtains maximum composite likelihood (MCL) estimates for ρ were obtained using the *Python* library *NLopt*. In addition to heRho (https://github.com/samebdon/heRho), we estimated the weighted mean of ρ across autosomes using the LDhelmet estimates of Booker *et al.* (2017) and Booker *et al.* (2021).

### Power analysis

To quantify how accurately CO and GC rates can be estimated, we performed a power analyses and parametric bootstrap on data simulated under the full model in msprime 1.0 (Baumdicker *et al.* 2022): we simulated 100 replicates for each chromosome under the MCL estimates obtained from the house mouse data (see Results). Each replicate consisted of 10 diploid samples assuming θ=0.071 (the observed heterozygosity), $\mu =5.410^{-9}$ (Uchimura *et al.* 2015), *L *=* *108.4 for all chromosomes. The rates of CO and GC were set to those inferred for each *M. m. castaneus* chromosome and the length of simulated sequence corresponded to the total length of intronic sequence analyzed for each chromosome (simulation code is available in the github repository).

We further use simulations to investigate the robustness to recombination rate variation and underlying demography. To assess the effect of recombination rate variation, we perform a simple comparison of 2 data sets: a control data set that combines 2 replicates with the same recombination rate and a test data set that combines 2 replicates with different rates, the average of which matches the control. For robustness to nonequilibrium population dynamics, we obtain estimates for a single long chromosome simulated under each scenario and evaluate heRho’s ability to estimate the underlying rates of recombination relative to mutation. See Supplementary Material 2 Demography for a full description of the models and a detailed analysis.

## Results

### Gene conversion explains the nonlinear relationship between estimates of ρ and physical distance

**Fig. 1.**
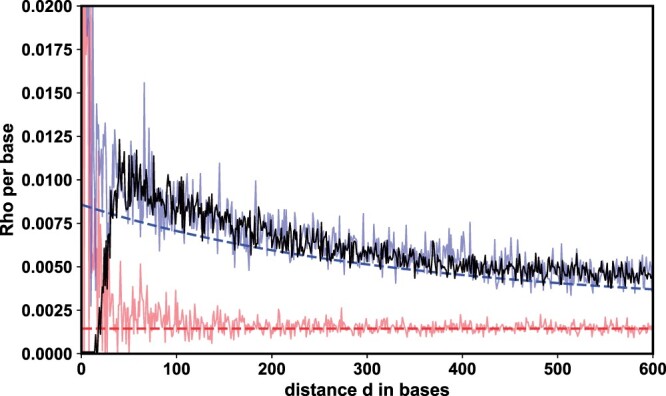
MCL estimates of ρ/bp at fixed distances *d* between pairs of sites; simulations with CO only (red), simulations with CO and GC (blue), and empirical data for *M. m. castanaeus* chromosome 19 (black). In each case, data was combined across a sample of 10 individuals. For simulations, θ=0.0071, κ=0.0014, γ=0.0036, and *L *=* *200. The dashed lines show the expectation under the corresponding model (Equations 1–4).

As a first step, we used Equations (1)–(4) to investigate how the per-base rate of recombination between pairs of sites depends on the distance between them (Fig. 1).We produce results for all chromosomes in the next section. Here, we provide a detailed and illustrative analysis of chromosome 19. Given a model that only includes CO events (red, dashed), we expect estimates of ρ/bp to be constant with respect to the distance between sites (red, dashed). However, when GC is included, nearby sites experience a higher per-base rate of recombination than pairs that are distant or unlinked (blue, dashed).

We find that estimates of ρ/bp based on a single replicate simulation either under a model with GC (solid blue) or without (solid red) follow the expected relationship with the distance between sites *d*. When inferring ρ/bp between pairs of loci at different distances *d* in the mouse data (Fig. 1, black line), the relationship between ρ/bp and *d* is similar to that seen for data simulated under a model of recombination that includes both CO and GC. We note that the pattern of distance-dependent ρ/bp is not exclusive to *M. m. castanaeus* but has been inferred previously for the ascidian *Cionia intestinalis* by Haubold *et al.* (2010) who suggest that GC may play a key role in shaping this signal.

Comparing the distance profiles of ρ/bp estimates between simulated and real data to each other and to analytic expectations (Fig. 1), we find 2 striking patterns:

First, in the real data, estimates of ρ/bp are close to zero for nearby pairs of sites and increase sharply over the first ≈50 bases. In contrast, while the accuracy and precision of ρ/bp estimates in simulated data is strongly dependent on *d* (i.e. there is high variability for estimates over short distances *d *<* *50), we find no similar monotonic increase in ρ/bp estimates over the first ≈50 bases. This discrepancy in estimates of ρ/bp in real versus simulated data suggests that over short distances ρ/bp estimates in the real data are biased downwards due to data quality/filtering effects: tightly linked polymorphisms are difficult to distinguish from complex mutations (e.g. indels) and/or are removed by so-called “best practices” variant calling/filtering approaches, skewing the observed values of *H*_0_, *H*_1_, and *H*_2_. This is compatible with the findings of Haubold *et al.* (2010) who have layered a sequencing error profile on coalescent simulations with CO only and shown that the noise generated by low-coverage data leads to a downward bias in estimates of ρ/bp over short distances.

Second, we find that estimates of ρ/bp are generally upwardly biased compared to expectations (compare solid and dashed lines in Fig. 1) due to simplifying assumptions about the mutational process: unlike real genomes which consist of a discrete number of bases, Equations (1)–(3) assume a continuous genome that evolves under the infinite sites mutation model. In that case, the occurrence of 2 mutations at a pair of sites always results in an *H*_2_ state. In contrast, under a finite-sites mutation model (which msprime assumes by default) a back mutation could generate an *H*_0_ state. Indeed, the resulting upward bias is observed only in simulations that assume finite sites (see Supplementary Fig. 1.1). The bias is strongest at short distances, where recombination is rare and the expected values of the *H_i_* are primarily governed by the mutational process. However, at greater distances, recombination primarily drives *H_i_* counts and estimates converge to the model predictions.

### Coestimating crossover and gene conversion rates

By decomposing recombination to distinguish CO and the distance-dependent effects of GC (Equation 5) and compositing the likelihood over the single-distance counts of *H_i_* (Equation 6), we may coestimate both the rates of CO (κ) and GC (γ) and the mean tract length *L*. However, there are 2 challenges in implementing this inference: (1) the noisiness of the data and the inaccuracy of the analytic results at short distances and (2) the inherent difficulty of coestimating strongly correlated parameters.

Given the biases over short distance in the real data, an obvious strategy is to introduce a minimum distance *d*_min_ in the CL (Equation 6). However, since most of the information to coestimate the rate of GC and the mean GC tract length is contained in short distances, there is a trade-off between minimizing bias and retaining information. Our exploration of this trade-off both in real and simulated data shows that parameter estimates are stable across a broad range of *d*_min_ values (Supplementary Fig. 1.2). To minimize the loss of information, we chose $d_{\text{min}}=100\;\text{bp}$ for all further analyses. Since genomes are finite and analysis is often restricted to a particular genomic partition, an upper distance threshold *d*_max_ is also unavoidable. To avoid biasing inference toward very long introns (which are selectively constrained), we limited the analysis to the first $d_{\text{max}}=1000\;\text{bp}$ of each intron.

**Fig. 2.**
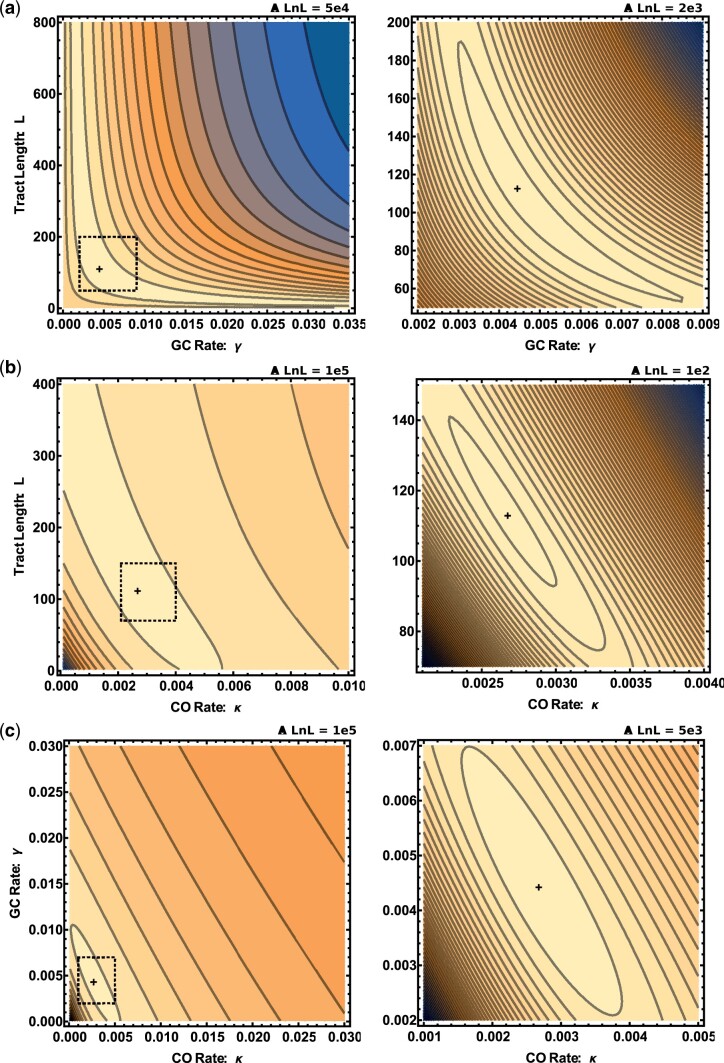
The CL surface for the rates of CO, GC, and the mean length of GC tracts for intronic data from *M. m. castaneus* chromosome 19. Each panel shows the 2-dimensional projection of the CL surface (ln CL increases from blue to yellow) under the global MCL estimates of parameters: κ=0.00267 (a), γ=0.0044 (b), and *L *=* *113.24 (c). For each panel, a broad parameter region is shown in the left plot, while the right plot focuses on the region near the optimum indicated by the dashed square. In all plots, the distance between contours is indicated at the top and the cross denotes the MCL estimate.

For the next step in our preliminary analysis, we asked whether sufficient information is retained to confidently coestimate the 3 recombination parameters. To do this, we focused on *M.m.castaneus* chromosome 19 using the distance thresholds $\left(d_{\text{min}},d_{\text{max}})=(100,1000\right)$. On this chromosome, heRho gives the following MCL estimates: κ=0.00267, γ=0.0044, and *L *=* *113.24. Examining the support, as measured by the logarithm of the composite likelihood (ln CL), surface around this maximum illustrates the challenge of coestimating *L* and κ. Although estimates are negatively correlated (Fig. 2b), we were positively surprised that it is not only possible to coestimate both parameters (the ln CL surface is smooth and contains a distinguishable optimum, Fig. 2), but that the estimates are indeed plausible, i.e. are compatible with direct, experimental estimates.

Less surprisingly, we observe that estimates for the GC rate γ and the mean tract length *L* are negatively correlated both with each other (Fig. 2a) and with estimates of the CO rate κ (Fig. 2, b and c). Given the degree to which parameter estimates are confounded and the fact that we have no biological reason to expect the length of GC tracts to vary between chromosomes, we chose to coestimate a global *L* and chromosome-specific GC and CO parameters in the subsequent analysis of the mouse data described below.

### The recombination profile of *M. m. castanaeus*

So far, we have obtained estimates separately for each chromosome. With only 3 parameters, optimization is very fast, and we use this to our advantage to identify an appropriate choice of *d*_min_ = 100 (Supplementary Fig. 1.2). Given this minimum distance, we estimate mean tract lengths between 100 and 200 bp. However, there is no biological reason to expect *L* to vary among chromosomes, and the strong correlation between *L* and γ may lead to biased results (Supplementary Fig. 1.5). To maximize the amount of data informing the choice of a genome-wide *L* and to obtain accurate estimates of chromosome-specific γ and κ, we coestimate all 19*2 + 1 parameters for the autosomes. We exclude the X chromosome from this global estimation because it experiences a different population history than the autosomes, and as a second step, we condition on the global estimate of *L* to obtain separate estimates of γ and κ for the X-chromosome.

**Fig. 3.**
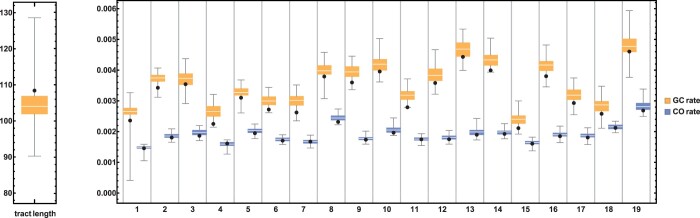
Recombination parameters coestimated for the 19 autosomes of *M. m. castanaeus* using data pooled across 10 individuals (black dots) and corresponding parametric bootstrap results from 100 replicate simulations. The per chromosome estimates of the GC rate (γ) and mean tract length are shown in yellow, estimates for the rate of CO (κ) in blue.

Our per autosome coestimates of the rates of CO (κ) and GC (γ) in *M. m. castaneus* (based on data from all 10 individuals, Fig. 3) range from 0.00145 to 0.00269 and 0.00211 to 0.00461, respectively. Assuming that the mean GC tract length is the same for all autosomes, our global MCL estimate for this parameter is 108 bases, which is within the range of previous direct estimates (≈10–300 for NCO and 200–1200 for CO GC tracts; Paigen *et al.* 2008; Mansai *et al.* 2011; Li *et al.* 2019; Cole *et al.* 2014). When restricting our analysis to a single individual, we obtain broadly concordant estimates of the CO rate and mean tract length, but with less data available, the GC rate estimates vary much more across chromosomes (Supplementary Fig. 1.3). Indeed, for simulations, increasing the sample size from one (Supplementary Fig. 1.4) to 10 individuals (Fig. 3) reduces the variance but not the bias of the results.

Our per autosome estimates recover several well-known, broad-scale patterns: First, as some GC events occur during CO, we expect the rates of CO and GC to be mechanistically and positively correlated, and this is indeed the case (Supplementary Fig. 1.5). Note that this signal contrasts with the negative correlation in the estimation error of both parameters (Fig. 2c) and therefore must reflect the underlying dynamics of meiotic recombination rather than any statistical artefact.

Second, as chromosomes have a minimum bound of map length at 50 cM due to obligate CO, we expect the CO rate per base to be negatively correlated with chromosome length. We recover this pattern (Supplementary Fig. 1.5) that is widely documented not only in mammals (Johnston *et al.* 2017), including humans (The International Genome Sequencing Consortium 2001), but also in flycatchers (Kawakami *et al.* 2014), yeast (Kaback *et al.* 1999), and butterflies (Martin *et al.* 2016). In contrast, we find that per chromosome estimates of the rate of GC are not significantly correlated with chromosome length (*P = *0.148) (Supplementary Fig. 1.5c). Since a high proportion of GC products are the result of non-CO recombination events, we do not expect GC rates to correlate significantly with patterns of chiasma formation.

As expected, the X chromosome carries less genetic variation than the autosomes (0.0038 vs 0.0071) and has a lower effective recombination rate: given the global estimate of the mean tract length *L *=* *108 bp, we estimate the X-chromosome CO rate and GC rate to be κ=0.0012 and γ=0.0006. Intriguingly, while the estimate of κ is generally concordant with that of the autosomes, we find that GC occurs at a rate 4–8 times lower on the X chromosome.

### Recombination rate variation

For many organisms, *M.musuculus* included (Booker *et al.* 2017), recombination rates even within a single chromosome can vary on a finer scale. How does this variation affect heRho’s estimates of recombination for aggregated data? Both for the CO-only and GC models, the estimated average κ is biased slightly downward for the mixed data set (Supplementary Fig. 1.6). For the GC model, the average estimates of both γ and *L* for the mixed data set match those of the control. Together, this suggests that we obtain a relatively accurate estimate of the average rate even though fine-scale variation is ignored.

### The effect of demography

Most population genetic methods for estimating recombination rates assume a simple panmictic population [with the exception of pyrho (Spence and Song 2019) which allows for step-wise changes in population size]. However, when naive to underlying demography, recombination rate estimates can be severely biased by nonequilibrium dynamics (Johnston and Cutler 2012; Kamm *et al.* 2016; Dapper and Payseur 2018; Samuk and Noor 2021). Because heRho estimates an effective rate of recombination ρ relative to diversity θ (κ/θ=k/μ and γ/θ=g/μ are constant), it may be robust to historic changes in population size. It is less clear, however, how robust the method is to more extreme population dynamics and population substructure. To address this, we perform simulations under 4 different demographic scenarios and compare the accuracy of recombination rate estimates (relative to the observed genetic diversity) to that under panmixia. We consider models for (1) a population bottleneck, (2) exponential growth, (3) admixture, and (4) 2-deme substructure, highlighting the main results here and providing a detailed analysis in the supporting information (Supplementary Demography).

As expected, heRho is relatively robust to the historic changes in population size under the bottleneck model. For a severe but brief bottleneck however, per-base recombination rate estimates ρ may be biased downward, particularly for site pairs with a small distance *d* between them, and under the GC model, γ is underestimated and *L* overestimated. In contrast to this, recent population growth under the exponential model leads to a substantial overestimate of the recombination rate. This bias diminishes as the within-pair distance between sites *d* increases, a pattern which may be mistaken for the effect of GC under a CO-only recombination model. Indeed, under the GC model, the method attributes this perceived excess of recombination to very high rates of GC events γ with very short tract lengths *L*.

The effects of population structure are more complicated. Admixture has very different effects on the estimates depending on the age of the admixture event. For both recombination models, older admixture events significantly bias ρ estimates downward, particularly at short distances *d*. In contrast, more recent admixture events lead to a significant overestimate of ρ for small values of *d* but a slight underestimate of ρ at greater distances. For the GC model, heRho obtains a consistent downward bias in CO rate κ across a range of admixture times but cannot reliably infer the GC rate γ or tract length *L*.

Similar to admixture, long-term population substructure prevents our method from obtaining estimates of the recombination rate. We consider a 2-deme model with equal population sizes of *N*_e_ and symmetric migration at rate *M*. We find that heRho performs well when migration rates are either very high (M≥≈1) or very low (M≤≈1e−4). If migration is rare, the ancestry of the sample reflects a single panmictic population with size *N*_e_. In contrast, with high migration rates, coalescence is equally likely to occur in either deme, and the ancestry resembles that of a panmictic population with size $2N_{e}$. However, between these 2 limits, the method generally fails to detect any recombination. To understand this, we used the framework of Lohse *et al.* (2011, 2016) to derive analytic expressions for the probability of the 2-locus heterozygosity states *H*_0_, *H*_1_, and *H*_2_ given two demes with symmetric migration (implemented in Mathematica Wolfram Research 2018, see Supplementary Notebook). As the migration rate increases from low values, there is a monotone transition in *H*_0_ and *H*_1_ from their respective probability in the $1N_{e}$ limit to that in the $2N_{e}$ limit. In contrast, as *M* increases from the $1N_{e}$ limit, *H*_2_ initially increases, surpassing that expected under panmixia, then decreases again to the $2N_{e}$ limit. As an excess of *H*_2_ relative to the observed genetic diversity is the informative signal of tight linkage under panmixia, the method infers that little to no recombination occurs.

## Discussion

A significant challenge in population genetics is to develop inference methods that are both efficient in extracting signals about population processes from sequence variation and simple, i.e. rely on a minimum number of assumptions. Given that high-coverage whole-genome data have become the norm, we now have the ability to study the fundamental forces of evolution, such as recombination, both at fine genomic scales and across a broad taxonomic range. We have developed a method for quantifying CO and GC from the distribution of heterozygous sites in small samples—even from individual diploid genomes.

### heRho’s strengths and weaknesses

As an extension of mlRho, our framework allows for more complete/realistic estimates of recombination from unphased data (Haubold *et al.* 2010). In general, one could argue that methods that do not rely on phased information (e.g. pyrho; Spence and Song 2019) are simpler and less error prone than those that do. For example, Booker *et al.* (2017) find that in the presence of switch errors, LDhelmet consistently overestimates the CO rate. Furthermore, by including homozygous states in the analysis, we garner sufficient information to coestimate CO and GC when data are restricted to short distances. As such, heRho can potentially generate a whole-genome annotation-specific recombination profile, even for small genomic partitions (e.g. first introns).

However, as demonstrated, our method heRho relies on large amounts of sequence data and is fundamentally limited by the frequency of the rarest 2-locus observation *H*_2_, which for any distance *d*, is of order *H*^2^. We therefore expect that it will not be possible to obtain estimates of GC and CO at finer genomic scales (say, in windows of 100 kb). While pooling observations across individuals increases the number of *H_i_* observations and reduces variance in the estimates, we expect many heterozygous sites to be shared among individuals, and thus the returns diminish quickly with sample size.

Although we find that that heRho is quite robust to heterogeneity when estimating an average recombination rate, our method does suffer from many of the same potential biases as other population genetic estimators of recombination. Given that we are assuming a neutrally evolving Wright–Fisher population of constant size, any demographic that affect LD will bias estimates of recombination obtained with heRho. Perhaps the most important question is whether there are processes that create false positive or false negative signals for the action of GC. We find that recent exponential growth and recent admixture both generate a false signature of GC in models with CO-only recombination, while in contrast, archaic admixture can obfuscate the true signals of GC and force heRho to falsely ascribe the effect of recombination primarily to CO. In the most extreme case, we found that both CO and GC are undetectable in the presence of strong population structure. As a result, great care is needed when interpreting estimates obtained from heRho, especially when the potentially confounding demographic histories are unknown. However, we also demonstrate that the method has the potential to overcome these limitations by extending the model to include demographic effects.

### Reconciling heRho’s recombination estimates for *M. m. castaneus* with LDHelmet

How do our estimates in *M. m. castaneus* compare to those obtained using LDHelmet and a CO-only recombination model? Coestimated under a model of GC, our genome-wide average of the CO rate per-base (0.00186) is approximately 5 times lower than the ρ estimates obtained by Booker *et al.* (2017, 2021) using LDhelmet (0.00924 and 0.0100, respectively, averaged across autosomes). If we instead compare the total recombination rate between any 2 adjacent bases, which corresponds to the upper bound of the recombination rate in our model (for *d *=* *1 eq. 5 reduces to ρ=κ+2γ), our estimate (0.00841) is much closer to that of the previous studies.

While this suggests that GC may contribute substantially to the ρ as estimated by Booker *et al.* (2017), this is unrealistic. LDHelmet uses longer-range SNP-only data, making it attune to the broader signal of CO and less sensitive to the very short-range effects of GC. Rather, the difference between the estimates likely reflects biology. Booker *et al.* (2017) estimates are obtained using data from large contiguous windows of the genome aggregated over all sites and genomic partitions which vary in proportion along the genome but will be dominated by intergenic sequence. Our estimates instead reflect the (per-chromosome) recombination profile specifically for the beginning of introns. Direct recombination estimates in humans suggest that the recombination rate in introns is lower than the genome-wide average (Myers *et al.* 2005). Furthermore, intron length is negatively correlated with recombination rates in some taxa (Comeron and Kreitman 2000), and our filtering strategy enriched for long introns. Note, however, that we cannot exclude the possibility that underlying demography has biased the results of one or both of these methods.

### Further applications and outlook

There are several potential avenues for further work, both empirically and analytically. In our anlaysis of *M.m. castaneus*, we infer a slightly lower CO rate on the X compared to autosomes as is expected for a hemizygous sex chromosome, however, the rate of GC we estimate is 4- to 8-times smaller than that of the autosomes. This may reflect a mechanistic difference in GC rates on the X, but it would be interesting to simulate recombination with both CO and GC on a sex-linked chromosome to see how this influences effective GC rate estimates. Furthermore, we have limited our analyses to long introns, but any genomic data partition for which pairwise heterozygosity can be accurately measured over a sufficient range of physical distances is suitable. It remains to be seen whether our method is informative about smaller genomic partitions such as centromeres and chromosomal inversions which differ from the genome-wide rates of recombination in systematic ways and where GC may occur but CO is restricted (Korunes and Noor 2017).

For further analytic work, first, it should be possible to relax the assumption of an infinite sites mutation model. While our analysis of the *M. m. castaneus* data reveals very small/tolerable biases (Fig. 3), basing estimates of GC and CO on more realistic mutation models might be important when analyzing more heterozygous genomes. Second, as a natural choice, we have assumed that loci are individual nucleotides. One could in principle extend the 2-locus inference to longer blocks of sequence and use the framework developed by Lohse *et al.* (2011) to base inference on the joint distribution of pairwise differences. However, this comes at the cost of introducing additional assumptions and biases. Third, it would be interesting to explore whether the machinery could be extended to 3 loci. If analogous analytic results for 3 loci are tractable, this would allow extracting substantially more signal and better estimate the rate and tract length of GC events from genomic data. Finally, we showed that the coalescent model and analytic expressions underlying heRho can be extended to include demography, and thus offers the potential, e.g. to coinfer migration rates in a structured population or to inform the method of a previously inferred demographic history.

## Data availability

The supporting information and the data, software code, scripts, and notebooks used to generate these results are available at https://github.com/samebdon/heRho.

Supplemental material is available at *GENETICS* online.

## Supplementary Material

iyac100_Supplementary_Fig_S1

iyac100_Supplementary_Fig_S2
