# Supplementary material for: Estimating the rates of crossover and gene conversion from individual genomes
Source: Genetics. 2022 Jun 30;222(1):iyac100. doi: 10.1093/genetics/iyac100 (PMC9434185; doi:10.1093/genetics/iyac100)
Supplement: iyac100_Supplementary_Fig_S1 [file iyac100_supplementary_fig_s1.pdf]

Estimating the rates of crossover and gene conversion from individual genomes

Supplementary Figures

Derek Setter, Sam Ebdon, Ben Jackson, Konrad Lohse*

*Institute of Evolutionary Biology, University of Edinburgh, Edinburgh, EH9 3FL, UK

June 17, 2022

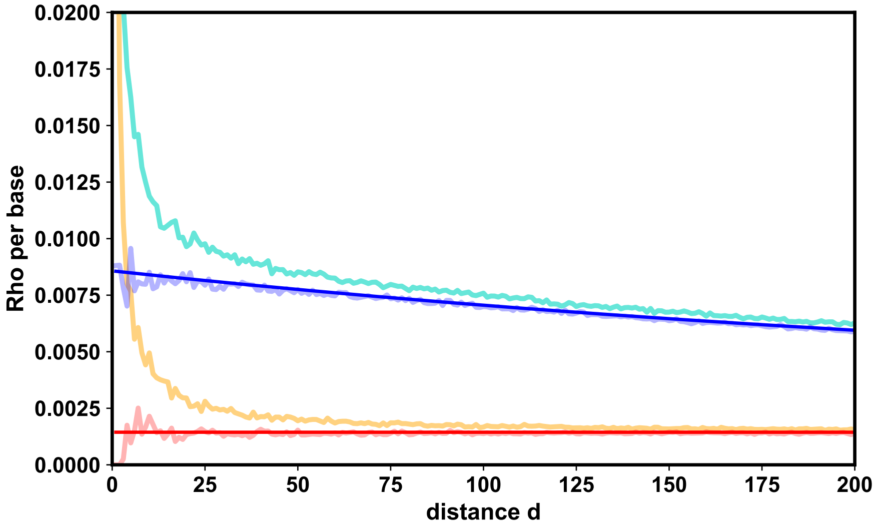

Figure S1.1: The bias of the estimator for discrete genomes. Here, we compare the analytic predictions for single-distance estimates of ρ/bp to those observed in simulations. The model predictions with and without GC are shown in dark blue and dark red, respectively. Light blue and light red show estimates from a coalescent model with a continuous genome, and the turquoise and orange lines to a discrete genome, correspondingly. Here, estimates were obtained from the combined data of 100 replicates under each simulated scenario. Parameters as in fig 1

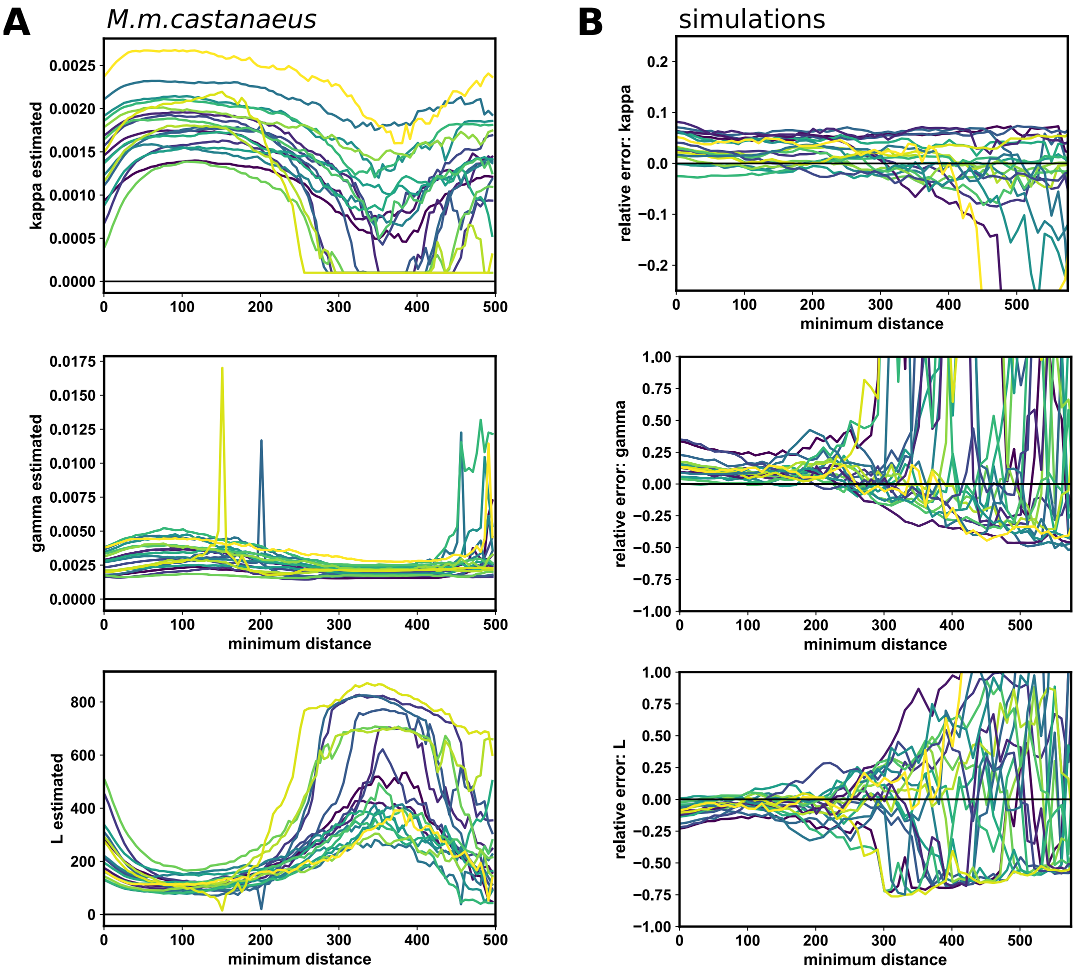

Figure S1.2: The effect of minimum distance on the per-autosome composite likelihood estimate of recombination. Panel A shows, from top to bottom, the estimated values of γ, κ, and L as a function of the minimum distance included in the likelihood calculation. Each indexed color corresponds to one chromosome, with chromosome 1 the darkest and chromosome 19 the lightest. Panel B shows the corresponding results obtained using the a single replicate of the simulations used for parametric boostrapping. The relative error in the estimated value, that is, the (observed - expected)/expected value, and the chromosomes are colored by index from the lowest (dark) to highest (light) recombination rate.

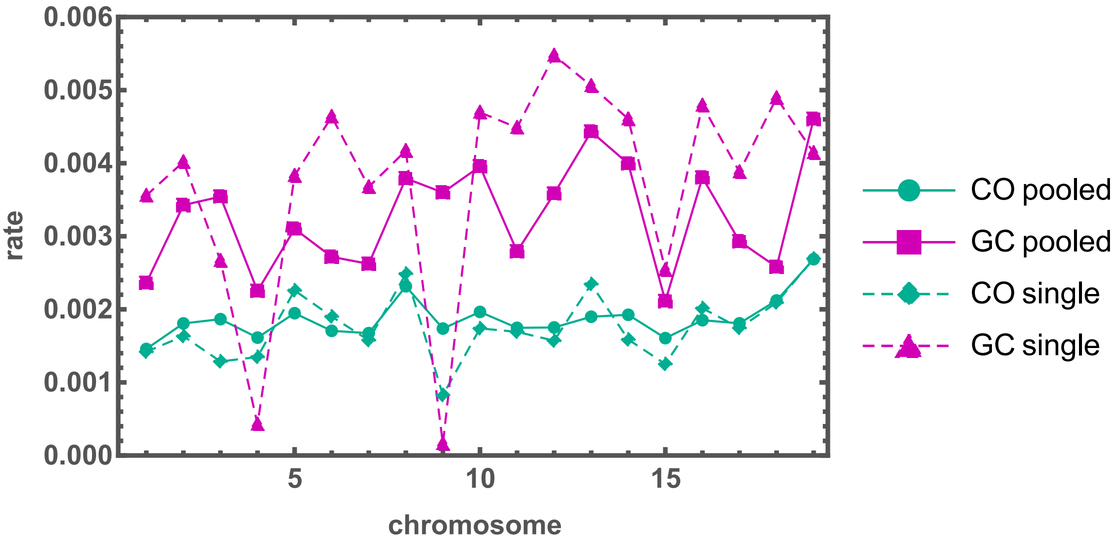

Figure S1.3: Comparison of the co-estimated CO rate (green) and GC rate (purple) *M. m. castaneus* for single-individual data (dashed) vs data pooled from ten individuals (solid). Each marker corresponds to a unique chromosome and estimate. The single- and multiple-individual estimates of the mean tract length were 107.8 and 108.4, respectively.

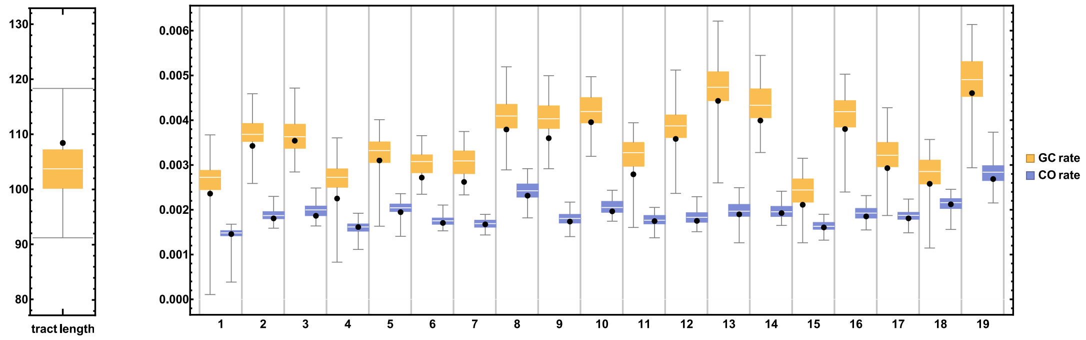

Figure S1.4: Bootstrapping results when data is limited to a single individual. The black dots correspond to the recombination parameters co-estimated for the autosomes using data pooled across ten *M. m. castanaeus* individuals (Fig. ??). Here, we randomly subsampled one individual from each of the simulation replicates. The per-chromosome GC rate and mean tract length estimates are shown in yellow, and the corresponding CO rate estimates are shown in blue.

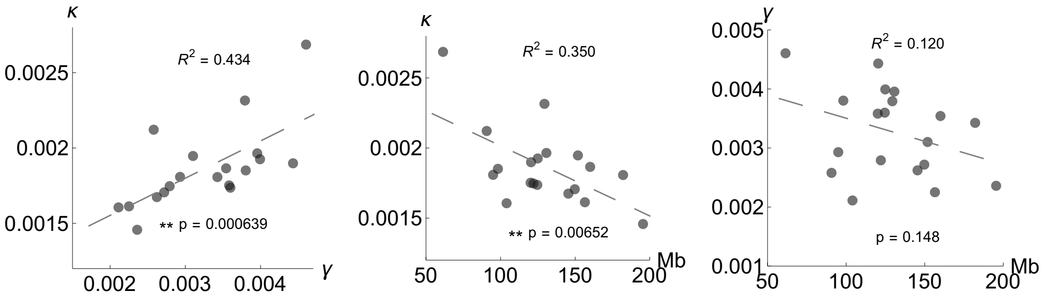

Figure S1.5: Left) Per chromosome estimates for the rates of CO and GC in *M. m. castaneus* are positively correlated; Center) Given that chromosomes have a roughly fixed map length, we expect ρ per base to correlate negatively with the physical length of chromosomes; Right) we find no analogous correlation between the rate of GC (γ) and chromosome length.

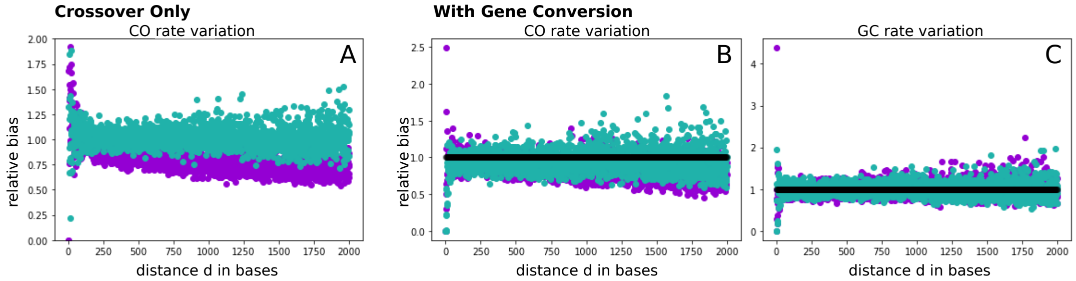

Figure S1.6: The effect of recombination rate variation on estimates obtained by `heRho`. Panel A shows the relative bias for the recombination rate under a crossover-only model. Teal shows estimates from combining two data sets simulated with the same CO rate $\kappa = 0.005$. Violet shows estimates from combining one data set with $\kappa = 0.002$ and one with $\kappa = 0.008$ such that the average remains the same. Panels B and C show the relative bias under a model that includes GC. In panel B, $\gamma = 0.005$ while κ varies as above. In panel C, $\kappa = 0.005$ while γ varies. Teal shows a combination of two simulations with the same $\gamma = 0.005$, and violet shows a combination of simulations, one with $\gamma = 0.002$ and one with $\gamma = 0.008$. The mean GC tract length was set at $L = 100$.
