# Supplementary material for: Estimating the rates of crossover and gene conversion from individual genomes
Source: Genetics. 2022 Jun 30;222(1):iyac100. doi: 10.1093/genetics/iyac100 (PMC9434185; doi:10.1093/genetics/iyac100)
Supplement: iyac100_Supplementary_Fig_S2 [file iyac100_supplementary_fig_s2.pdf]

Estimating the rates of crossover and gene conversion from individual genomes

Supporting Information: Demography

Derek Setter, Sam Ebdon, Ben Jackson, Konrad Lohse*

*Institute of Evolutionary Biology, University of Edinburgh, Edinburgh, EH9 3FL, UK

June 17, 2022

Demography

Here we investigate the effect of demography on the recombination rates inferred by `heRho`, both for a crossover(CO)-only model and a model of crossover and gene conversion (GC). We explore four demographic scenarios: (i) a population bottleneck, (ii) exponential growth, (iii) recent admixture, and (iv) a structured population.

For each combination of recombination model and demographic scenario, we simulate a single 50Mb chromosome, sample a single diploid individual, and estimate both the recombination rate ρ per-base and the composite estimate of the CO rate κ, GC rate γ, and tract length L. Simulations were run using `msprime 1.0.2` [Baumdicker et al., 2022] and *M. musculus*-like parameters: populations size $N_e = 328,704$, per-base rates of mutation $\mu = 5e-9$, CO $k = 1.293e-9$, and GC $g = 2.662e-9$, and a mean conversion tract-length of $L = 108$ base pairs.

Note that `heRho` co-estimates the population-scaled rates of recombination ρ and mutation θ under a model of constant population size. In order to compare performance across demographic scenarios in which N_e varies, we introduce a standardized measure that we call the *relative bias*. We define the *relative bias* $\beta_d(\rho)$ of the per-base recombination rate between sites separated by a given distance d as the ratio of the estimated value of ρ/θ to the expected value of ρ/θ under the corresponding model, $\beta_d(\rho) = \frac{\rho_{est}}{\theta_{est}} / \frac{\rho_{exp}}{\theta_{exp}}$. This ratio-of-ratios allows us to compare models with and without gene conversion and informs us how well `heRho` performs: values lower that one indicate an underestimate while values greater than one indicate an overestimate of the relative recombination rate. Similarly, for composite estimates, we compare the estimated ratio of κ/θ and γ/θ to the expected ratios of $\kappa_{exp}/\theta_{exp} = k/\mu = 0.240$ and $\gamma_{exp}/\theta_{exp} = g/\mu = 0.493$ to measure the relative bias in the CO rate $\beta(\kappa)$ and GC rate $\beta(\gamma)$, respectively. Because the gene conversion tract length does not scale with the population size, we simply record the estimated mean length L for each scenario.

Population Bottleneck

We consider a population that has undergone a bottleneck in population size. Looking past-ward and measuring time on the coalescent scale, we consider a bottleneck which occurred $T_{bottle} = 0.5$ $(2N_e)$ generations ago. The population size is reduced from N_e to N_b for a duration of time $T_{duration}$, after which, it returns to the ancestral population size of N_e. We consider three combination of parameters for the duration and severity of the bottleneck: a strong, intermediate and weak bottleneck [corresponding to $(T_{duration}, N_b) = (0.1, 0.1 * N_e)$ $(0.5, 0.5 * N_e)$ and $(0.9, 0.9 * N_e)$ respectively]. Note that these parameter combinations are chosen such that the pairwise probability of coalescing during the bottleneck is the same in all three scenarios 0.384.

We find that **heRho** is quite robust to the effect of population size bottlenecks (Fig. S2.1), though very short-lived and strong bottlenecks may cause no recombination to be detected among very tightly linked site pairs S2.1 and slightly biases per-base ρ estimates downward at greater distances, particularly in the model with gene conversion. Indeed, for simulations with gene conversion, a strong bottleneck induces a bias downward in γ and upward in mean tract length L (Table S2.1). Otherwise, the composite estimates are generally close to the true values.

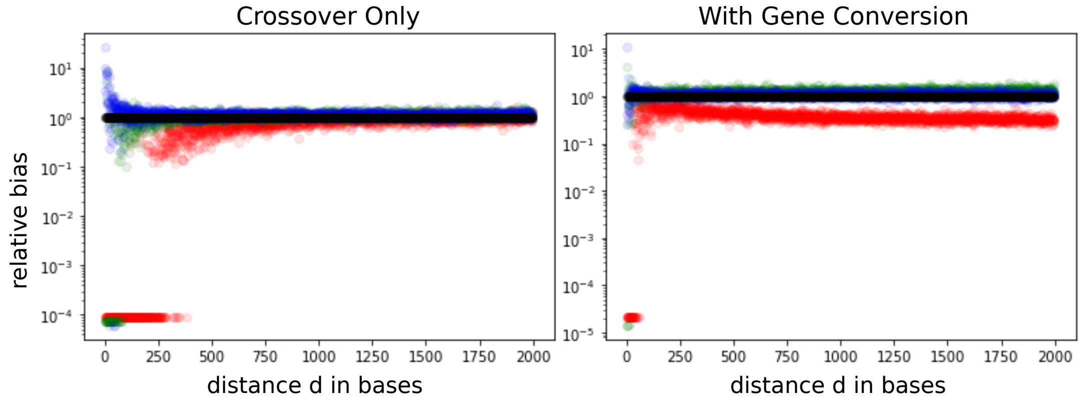

Figure S2.1: The effect of a population bottleneck on recombination estimates. We plot the *relative bias*: $\beta_d(\rho) = \frac{\rho_{est}}{\theta_{est}} / \frac{\rho_{exp}}{\theta_{exp}}$ for various distances between site pairs d. The left panel shows estimates for the CO-only model, the right for a model with GC for strong (red), intermediate (green) and weak (blue) bottleneck. These correspond to $(T_{duration}, N_b) = (0.1, 0.1 * N_e)$ $(0.5,\ 0.5 * N_e)$ and $(0.9, 0.9 * N_e)$ respectively.

Table S2.1: Bias in composite estimates under the bottleneck model with gene conversion. Here we show the estimated mean tract length L and the *relative bias* in the estimated rates of CO $\beta(\kappa) = \frac{\kappa_{est}}{\theta_{est}} / \frac{\kappa_{exp}}{\theta_{exp}}$ and GC $\beta(\gamma) = \frac{\gamma est}{\theta_{est}} / \frac{\gamma exp}{\theta_{exp}}$ for strong, intermediate, and weak bottlenecks. The color denotes the corresponding data set from the right panel of Fig. S2.1

color	$T_{duration}$	N_b/N_e	$B(\kappa)$	$B(\gamma)$	L
red	0.1	0.1	1.16	0.34	254
green	0.5	0.5	1.05	1.15	98
blue	0.9	0.9	1.32	1.02	113

Exponential Growth

We consider a population that has experienced exponential growth to its current N_e from an ancestral population of size $N_a = 1/10N_e$ and vary the time T_g since the exponential growth began: $T_g = \{0.05, 0.5, 2.0\}$, corresponding to scaled growth rates of $\{92.1, 4.6, 1.15\}$, respectively.

Exponential growth befuddles **heRho**, both in a CO-only model and a model with GC (Fig. S2.2), causing a strong upward bias in per-base ρ, particularly over short distances d. Composite estimates of the recombination parameters also show strong biases. With very recent growth, little to no CO is detected, and the model attributes recombination to a high rate of GC with very short tracts (Table S2.2). With slower growth, the estimated CO rate instead shows a slight upward bias. However, estimates of the GC rate are still strongly biased upward and tract lengths downward.

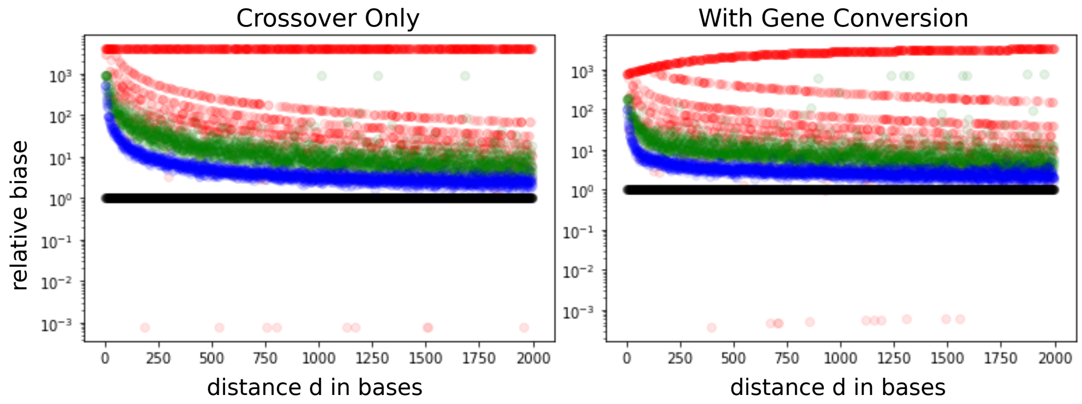

Figure S2.2: The effect of exponential growth on recombination estimates. We plot the *relative bias* (see Fig. S2.1) for various site-pair distances d. The left panel shows the CO-only model, the right a model with GC. The time when exponential growth started is $T_g = 0.05$ (red), $T_g = 0.5$ (green), and $T_g = 2.0$ (blue).

Table S2.2: Bias in composite estimates under the exponential growth model with GC. Here we show the estimated mean tract length L and the *relative bias* (see Fig. S2.1) for CO $B(\kappa)$ and GC $B(\gamma)$ and estimated GC tract length for varying duration T_g of the exponential growth phase and an ancestral population of one-tenth the current population size. The color denotes the corresponding data set from the right panel of Table S2.1

color	T_g	N_a/N_e	$B(\kappa)$	$B(\gamma)$	L
red	0.05	0.1	0.0008	1938	12
green	0.5	0.1	2.23	182	10
blue	2.0	0.1	1.66	52	10

Admixture

We consider an ancestral population of size N_e that splits into two isolated populations, each of size N_e, that remain isolated for a duration $T_{div} = 2.0$ $(\times 2N_e)$ generations. After this period of isolation, the current population of size N_e is created from a 50:50 admixture event. We consider different times since the admixture event $T_{mix} = \{0.01, 0.1, 0.5, 0.75, 1.25, 2.0\}$; i.e. the total time since the divergence occurred is $T_{div} + T_{mix}$.

For both CO-only and GC models, admixture has the same intriguing effect on the per-base ρ estimated for site pairs separated by varying distances d: for recent admixture (Fig. S2.3: red, green, and blue), ρ estimates are biased upward over short distances d. This bias diminishes as d increases, eventually becoming slightly biased downward. In contrast, when admixture is old (Fig. S2.3: orange, yellow, black), per-base ρ estimates are strongly biased downward, and over very short distances no recombination is detectable. This bias diminishes with increasing distance d, but does not change in direction. We speculate that for small d, admixture biases ρ estimates upward when $T_{mix} < \ln(2) \approx 0.69$, for which the probability of coalescing before the admixture event is less than 1/2, while $T_{mix} > \ln(2)$, results in a downward bias.

For the GC model, the estimate of κ was only slightly biased downward, irrespective of the time since admixture T_{mix} (Table S2.3). In contrast, GC rates and the mean tract length L are very poorly estimated. For very recent admixture, `heRho` identifies very high GC rates and short mean tract lengths (L), while old admixture leads to a false signal of low GC rates and long L.

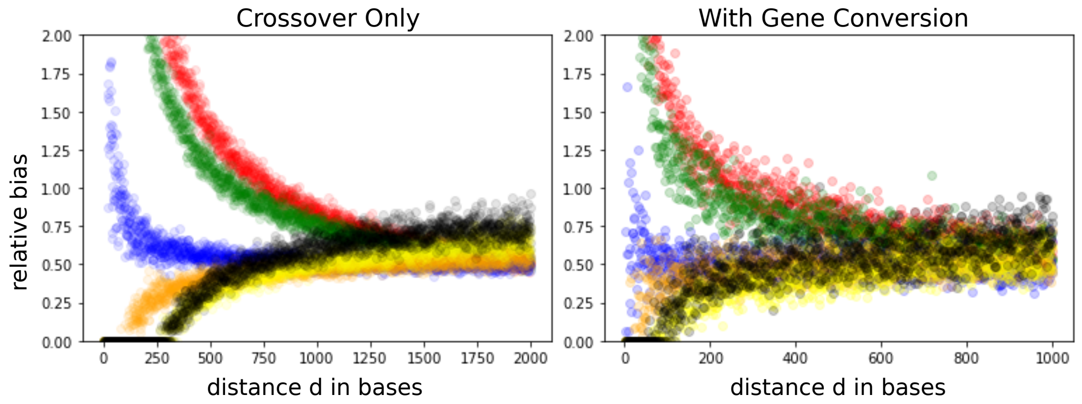

Figure S2.3: The effect of admixture on recombination estimates. We plot the *relative bias* (see Fig. S2.1) for various site-pair distances d. The left panel shows the CO-only model, the right a model with GC. Divergence always occurs for a duration of $T_{div} = 2.0$ ($\times 2N_e$ generations), while the time since admixture varies: $T_{mix} = 0.01$ (red), 0.1 (green), 0.5 (blue), 0.75 (orange), 1.25 (yellow) and 2.0 (black).

Table S2.3: Bias in composite estimates under the admixture model with gene conversion. Here we show the estimated mean tract length L and the *relative bias* (Table S2.1) for CO $B(\kappa)$ and GC $B(\gamma)$ and the estimated GC tract length for varying times since admixture ocurred T_{mix}. The color denotes the corresponding data set from the right panel of Fig. S2.3

color	T_{div}	T_{mix}	$B(\kappa)$	$B(\gamma)$	L
red	2.0	0.01	0.33	16	10
green	2.0	0.1	0.4	11	10
blue	2.0	0.5	0.57	1.03	156
orange	2.0	0.75	0.41	0.18	415
yellow	2.0	1.25	0.83	2e-5	532
black	2.0	2.0	0.335	0.21	2000

Structured Population

In this scenario, we consider a population sub-divided into two demes, each of size N_e, experiencing symmetric migration at varying (coalescent scaled) rates M. We sample a single diploid individual from one sub-population. We first focus on four values of the migration rate $M = \{5e-5, 5e-3, 5e-1, 5e+1\}$.

We find that estimates of ρ per-base relative to θ are very accurate when migration rates are either low or high, both for the CO-only and the GC recombination models (Fig. S2.4, red and orange). In contrast, intermediate rates of M lead to a strong downward bias (blue) and may obscure the signal of recombination altogether (green). This is echoed in the composite likelihood estimates under the GC model (Table S2.4): accurate estimates for κ, γ and L are obtained with low or high migration rates, while all parameters are significantly underestimated at intermediate migration rates.

To understand this better, we investigated per-base ρ estimates for a more-detailed set of migration rates M both for the CO-only model (Fig. S2.5) and the GC model (Fig. S2.6). We consistently estimate very low or negligible recombination rates for M between $5e-4$ and $5e-1$. When we look at the ρ estimated for large d (where GC has only a weak effect and ρ per-base is dominated by the CO rate), we observe twice as much recombination (not scaled by θ) at high migration rates (e.g. $M = 5e{+}1$) relative to that observed for low migration rates (e.g. $M = 5e{-}6$). This matches our expectation that when migration is sufficiently rare, the population dynamics resemble those of a single population with size N_e, while at high migration rates, the population is only weakly structured and behaves like a single population of size $2N_e$.

Why, then, does `heRho` fail to detect recombination in populations with appreciable substructure? To address this question, we use the framework of Lohse et al. [2011, 2016] to obtain the generating function for a two-locus, two-deme coalescent model with symmetric migration and recombination. We derive analytic expressions for the expected probability of the two-locus heterozygosity measures $H0$, $H1$, and $H2$ used to estimate ρ (using Mathematica [Inc.], see S2 Notebook). As expected, the predictions under the two-deme model (Fig. S2.7, black) converge to the one-deme model with population size N_e at low migration rates M (red), while for large M, converging to the one-deme model with population size $2N_e$ (blue). For $H0$, the transition between these two limits is monotone in M. Although $H1$ decreases very slightly from $M = 0$ to $M \approx 1e - 4$, over the range of M with appreciable sub-structure (approximately 1e-3 to 1.0), $H1$ increases monotonically. In contrast, over this range, the effect of migration on $H2$ is non-monotone. Intermediate migration rates generate tracts of heterozygosity causing an excess of double-heterozygous site pairs relative to both the low and high M limits. It is the over-abundance of $H0$ and $H2$ types that indicates strong linkage between site pairs, and naive to the effect of underlying substructure, `heRho` misinterprets this as a signal of little to no recombination.

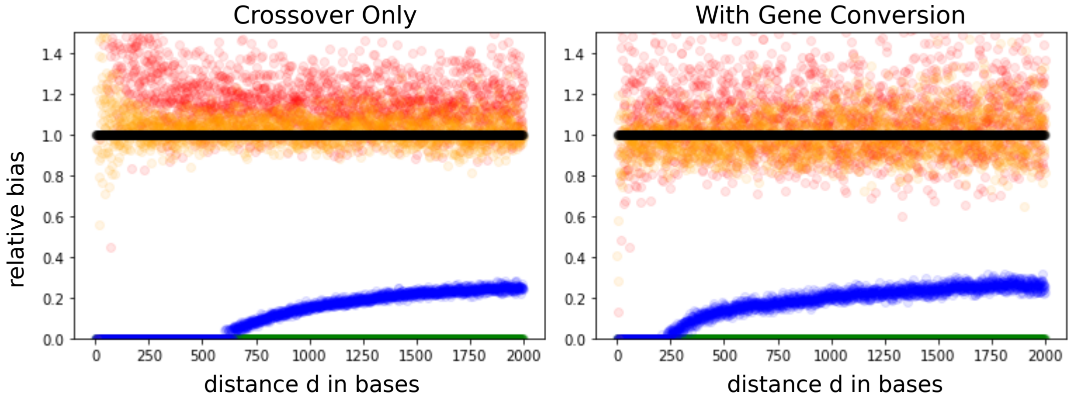

Figure S2.4: The effect of population structure on recombination estimates. We plot the *relative bias* (see Fig. S2.1) for various site-pair distances d. The left panel shows the CO-only model, the right a model with GC. We consider two demes of size N_e with symmetric migration at varying rates: $M = 5e-5$ in red, $5e-3$ in green, $5e-1$ in blue, and $5e+1$ in orange.

Table S2.4: Bias in composite estimates under the structure model with GC. Here we show the estimated mean tract length L and the *relative bias* (see Table S2.1) for CO $B(\kappa)$ and GC $B(\gamma)$ and estimated GC tract length for varying migration rates M. The color denotes the corresponding data set from the right panel of Fig. S2.4

color	M	$B(\kappa)$	$B(\gamma)$	L
red	5e-5	1.5	1.1	104
green	5e-3	4e-5	2e-5	10
blue	5e-1	0.29	1.5e-5	10
orange	5e+1	1.02	0.96	102

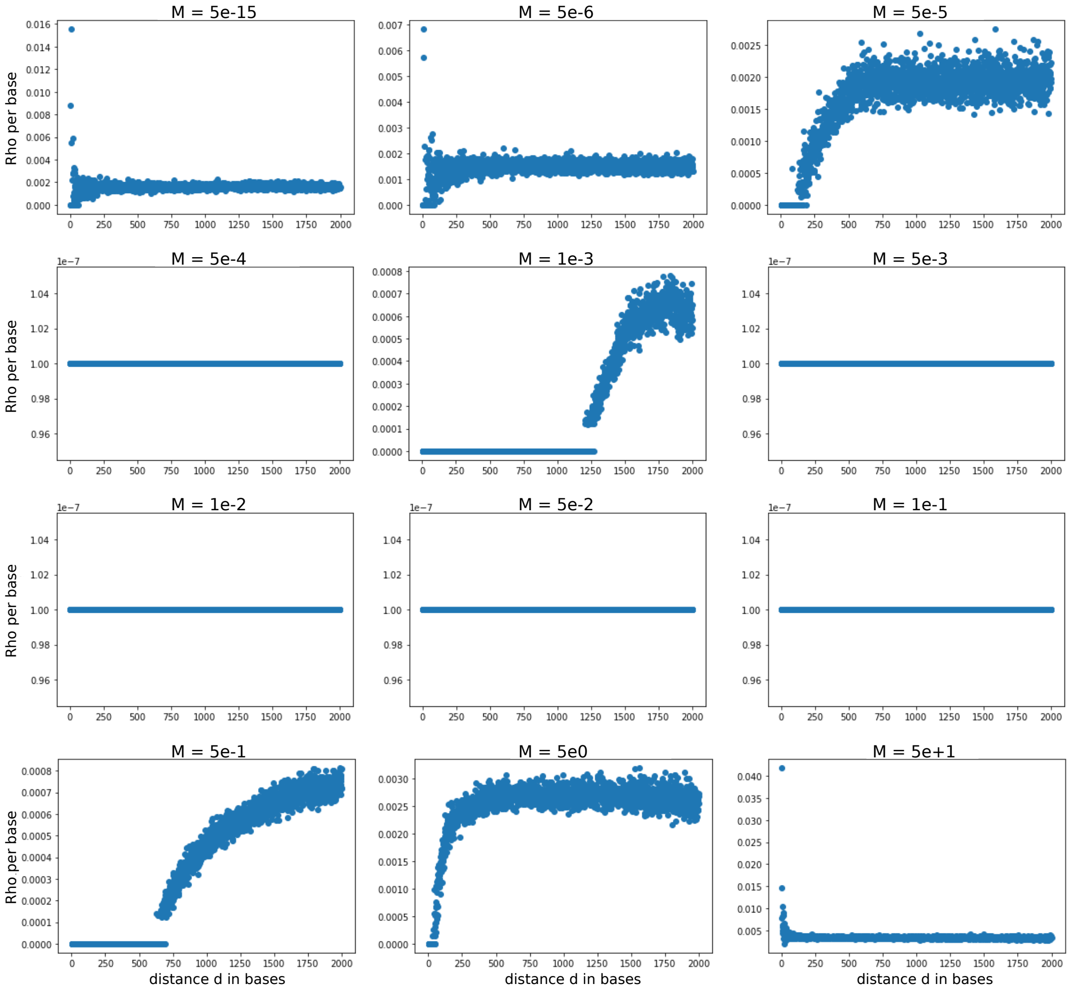

Figure S2.5: CO only model: Per-base ρ estimates as a function of site-pair distance d for varying migration rates M under the two-deme model with symmetric migration. Each panel shows the results for a single iteration simulated with the migration rate M denoted above it. Here, recombination can occur through CO only. Note that the y-axis in some plots is scaled by 1e-7. This is the lower bound of the parameter space used in the optimization procedure so represents an estimated recombination rate of 0.

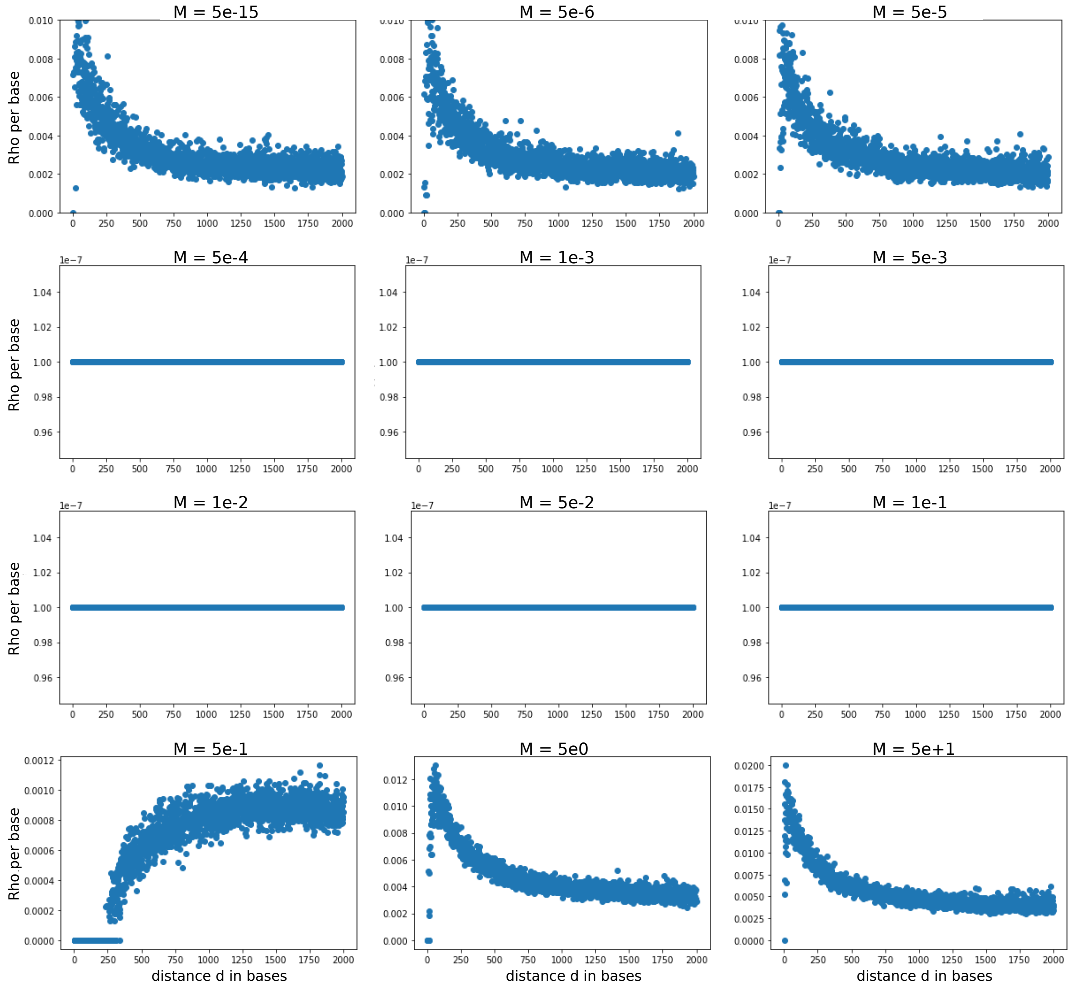

Figure S2.6: Per-base ρ estimates as a function of site-pair distance d for varying migration rates M under the two-deme model with symmetric migration and CO-only recombination. Each panel shows the results for a single iteration simulated with the migration rate M denoted above it. Here, recombination can occur through CO and GC. Note that the y-axis in some plots is scaled by 1e-7. This is the lower bound of the parameter space used in the optimization procedure so represents an estimated recombination rate of 0

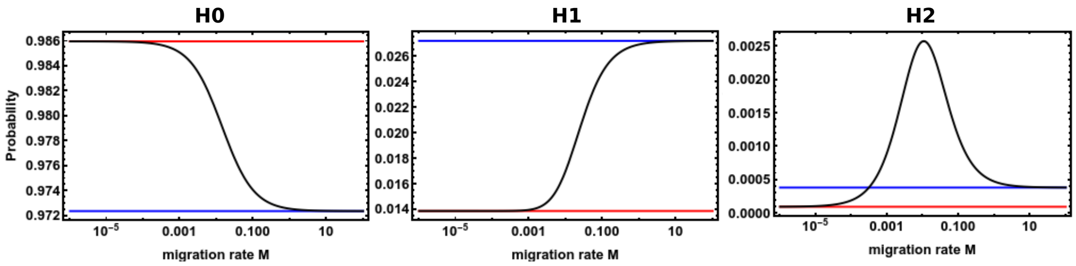

Figure S2.7: The effect of migration on expected two-locus heterozygosity probabilities. Here we plot the probability of $H0$, $H1$, and $H2$ as a function of the migration rate M. Red and blue lines show the expectations under panmixia for population sizes $1N_e$ and $2N_e$ respectively. The black lines shows the probabilities obtained under the two-deme model.

References

Franz Baumdicker, Gertjan Bisschop, Daniel Goldstein, Graham Gower, Aaron P Ragsdale, Georgia Tsambos, Sha Zhu, Bjarki Eldon, E Castedo Ellerman, Jared G Galloway, Ariella L Gladstein, Gregor Gorjanc, Bing Guo, Ben Jeffery, Warren W Kretzschumar, Konrad Lohse, Michael Matschiner, Dominic Nelson, Nathaniel S Pope, Consuelo D Quinto-Cortés, Murillo F Rodrigues, Kumar Saunack, Thibaut Sellinger, Kevin Thornton, Hugo van Kemenade, Anthony W Wohns, Yan Wong, Simon Gravel, Andrew D Kern, Jere Koskela, Peter L Ralph, and Jerome Kelleher. Efficient ancestry and mutation simulation with msprime 1.0. *Genetics*, 220(3), 3 2022. ISSN 19432631. doi: 10.1093/GENETICS/IYAB229. URL `https://academic.oup.com/genetics/article/220/3/iyab229/6460344`.

K. Lohse, R. J. Harrison, and N. H. Barton. A general method for calculating likelihoods under the coalescent process. *Genetics*, 189(3):977–987, 11 2011. ISSN 00166731. doi: 10.1534/genetics.111.129569.

Konrad Lohse, Martin Chmelik, Simon H. Martin, and Nicholas H. Barton. Efficient strategies for calculating blockwise likelihoods under the coalescent. *Genetics*, 202(2):775–786, 2 2016. ISSN 19432631. doi: 10.1534/genetics.115.183814.

Wolfram Research, Inc. Mathematica, Version 12.1. Champaign, IL, 2020.
